# 乙醇含量对黄酒挥发性组分检测的影响

**DOI:** 10.3724/SP.J.1123.2022.07018

**Published:** 2023-05-08

**Authors:** Jian HU, Yuanyuan HUANG, Shuangping LIU, Jian MAO

**Affiliations:** 1.江南大学食品学院,粮食发酵与食品生物制造国家工程研究中心,江苏无锡214122; 1. National Engineering Research Center of Cereal Fermentation and Food Biomanufacturing, School of Food Science and Technology, Jiangnan University, Wuxi 214122, China; 2.上海金枫酒业股份有限公司,上海201501; 2. Shanghai Jinfeng Wine Co., Ltd., Shanghai 201501, China

**Keywords:** 顶空-气相色谱法, 乙醇, 基质效应, 挥发性组分, 定量检测, headspace-gas chromatography (HS-GC), ethanol, matrix effect, volatile components, quantitative detection

## Abstract

黄酒是风味独特的发酵酒,酒中组分复杂,且不同种类黄酒中乙醇含量差异较大。通过顶空-气相色谱法(HS-GC)测量不同乙醇含量的黄酒中挥发性组分色谱峰面积的变化,分析了乙醇引起的基质效应对不同挥发性组分定量检测的影响。在乙醇含量为10%~19% vol(酒精度)的黄酒中,16种挥发性组分(仲丁醇、正丙醇、异丁醇、正丁醇、异戊醇、*β*-苯乙醇、乙醛、异戊醛、苯甲醛、甲酸乙酯、乙酸乙酯、乙酸异丁酯、乙酸异戊酯、己酸乙酯、乳酸乙酯、丁二酸二乙酯)的峰面积与乙醇含量呈线性负相关。酒中乙醇含量的升高改变了其他大部分微量挥发性组分的气液平衡。乙缩醛的峰面积与乙醇含量呈线性正相关,两者在乙醇浓度变化过程中发生化学反应。甲醇、糠醛和乙酸的峰面积受乙醇含量的影响较小。色谱峰面积受乙醇含量的影响系数为-12.4%~4.9%,不同挥发性组分的蒸汽压及气液平衡受到乙醇基质效应的影响程度不同。将不同黄酒样品调整至同一酒精度后,不同样品中挥发性组分在溶液中的含量与色谱峰总面积成正比,乙醇引起的基质效应对检测结果的干扰得到有效降低。研究人员在使用基于气液平衡等原理的前处理方法开展风味组分定量检测时,应将不同黄酒样品调整至相同的酒精度,才能有效控制乙醇含量差异引起的基质效应,实现准确的定量分析。

## Abstract (en)

Huangjiu (Chinese rice wine) is a traditional Chinese fermented wine with a unique flavor. The components of this wine are complex, and the ethanol content of different Huangjiu preparations varies greatly. In this study, changes in the chromatographic peak areas of the volatile components of Huangjiu samples with different ethanol contents were measured using headspace-gas chromatography (HS-GC). The influence of ethanol on the quantitative detection of different volatile components of Huangjiu at gas-liquid equilibrium was also analyzed. When the ethanol content of Huangjiu was in the range of 10%-19% vol, the peak areas of 16 volatile components (i. e., *sec*-butanol, *n*-propanol, isobutanol, *n*-butanol, isoamyl alcohol, *β*-phenyl-ethanol, acetaldehyde, isovaleraldehyde, benzaldehyde, ethyl formate, ethyl acetate, isobutyl acetate, isoamyl acetate, ethyl hexanoate, ethyl lactate, and diethyl succinate) were negatively correlated with the ethanol content. Increases in the ethanol content of the liquor changed the gas-liquid equilibrium of most other trace volatile components. In addition, only the peak area of acetal was positively correlated with ethanol content. The content of acetal in Huangjiu was affected by the alcohol content, and its decomposition reaction occurred along with the dilution process. The influence coefficient of ethanol content on the peak area of the above compounds ranged from -12.4% to 4.9%. The vapor pressure of most volatile components decreased with increasing ethanol content, and different components were affected in different ways. Compared with those of other components, the peak areas of methanol, furfural, and acetic acid were less affected by the ethanol content. These components were also affected by other factors, such as ionization and chemical reactions occurring during the dilution process. When different wine samples were adjusted to the same ethanol content, the concentration of volatile components in these samples became proportional to the total chromatographic peak area and the influence of the matrix effect of ethanol on the quantitative analysis was effectively eliminated. Thus, when researchers use pretreatment methods based on the principle of gas-liquid balance to carry out the quantitative detection of flavor components, they should adjust different rice wine samples to the same alcohol content to effectively control the matrix effect caused by differences in ethanol content and achieve accurate quantitative analysis.

黄酒是中国传统发酵酒,受产地、工艺的影响,不同黄酒的组分和风味差异较大^[1]^。在黄酒的挥发性风味组分中,体积分数最高的是乙醇(6%~25% vol(酒精度)),乙醇通常采用蒸馏等前处理后用密度瓶法检测^[2]^。而正丁醇、乳酸乙酯等醇类和酯类挥发性风味组分的含量一般都低于1 g/L^[3]^,浓度较低,需通过气相色谱等手段进行准确检测。目前,中国已经实施了2项轻工行业标准,分别是QB/T 4708-2014和QB/T 4709-2014^[4,5]^。这两项标准采用同一种前处理方法,都是在黄酒中添加氯化钠及内标叔戊醇,然后采用顶空-气相色谱法(HS-GC)测定挥发性组分中醇类和酯类物质的含量。同时,采用标准物质增量法计算酒样的校正因子,减少基质效应。除HS-GC已经在黄酒行业普遍应用外,顶空固相微萃取-气相色谱-质谱法(HS-SPME-GC-MS)也经常被用于微量风味组分的检测。Chen等^[6]^先将黄酒用水稀释到6% vol后,再添加氯化钠及内标2-辛醇,并采用HS-SPME-GC-MS定量分析黄酒中挥发性香气物质的含量。郭伟灵等^[7]^将红曲黄酒用水稀释10倍处理后,再用HS-SPME-GC-MS定量检测香气物质的含量。王程成等^[8]^采用液液微萃取结合GC-MS,建立了黄酒中“焦糖香”特征香气物质葫芦巴内酯的分析方法。陈子凡等^[9]^采用直接进样GC-MS,分析了9种醇类化合物的含量。周慧敏等^[10]^利用具有8种气体传感器的电子鼻系统,通过测定挥发性组分含量间接实现黄酒类型和总糖含量的快速预测。此外,在挥发性风味组分定性研究方面,惠国华等^[11]^通过电子鼻系统评价不同酒精度黄酒中的特征性香气,于海燕等^[12]^通过气相色谱-嗅闻法直接分析黄酒中酯类组分间的香气协同作用。以上大部分研究方法,都需要将液体样品在一定温度条件下进行气液平衡后,通过检测气体中的组分含量间接得到液体中组分的含量。

由于发酵酒的组分比较复杂,基质效应会严重干扰酒中组分的准确定量。Higashikawa等^[13]^研究了基质效应在HS-SPME-GC-MS分析中的影响,基质中含有少量极性组分会干扰醇和酮等极性化合物的检测。Qin等^[14]^通过顶空单滴微萃取结合气相色谱法检测酒中甲醇含量,通过单滴微萃取控制红酒的基质效应。Pizarro等^[15]^通过顶空固相微萃取结合气相色谱-串联质谱检测技术,用于消除基质效应对酒中卤代苯甲醚和挥发酚的检测干扰。黄酒的组分同样复杂,含有多种糖、有机酸、氨基酸等难挥发性组分和醇、酯、醛等挥发性组分^[16]^。而以上针对黄酒的研究中,无论是引用行业标准进行分析,还是自行开发特定分析方法,大部分研究者并未明确关注黄酒基质效应对定量结果的影响。顶空检测方法的原理是依据亨利定律,适合于理想稀溶液,而乙醇与水混合组成溶液时,存在体积变化及热效应,属于非理想溶液。因此,当乙醇含量变化时,对其他溶质的蒸汽压产生影响,改变气液平衡,使得检测结果发生偏差。根据刘晓黎等^[17]^的研究,不同溶剂会对溶质的峰面积响应值有影响。黄酒中乙醇含量差异较大,最低的仅有6% vol,而最高的香雪酒有25% vol。黄酒中乙醇的含量及变化范围远高于葡萄酒、啤酒等发酵酒,对分析方法的影响也更大。酒样可以通过大比例稀释在一定程度上降低基质效应,但也同时降低了分析方法的检测灵敏度。

本研究比较了不同乙醇含量的黄酒在顶空条件下色谱峰面积的变化,分析乙醇产生的基质效应对不同挥发性组分定量检测的影响,从而加强风味研究人员对黄酒基质效应的关注。同时,通过调整黄酒酒精度,控制乙醇含量差异带来的基质效应,更准确地测定不同黄酒样品中的挥发性组分。

## 1 实验部分

### 1.1 仪器、材料与试剂

6890N气相色谱仪(美国Agilent公司),配有DB-WAX色谱柱(60 m×0.32 mm×0.25 μm)和HP-7694E型顶空进样器;Alcolyzer Wine酒精分析仪(奥地利安东帕公司),配有DMA 4100M密度计;AL204型电子分析天平(瑞士梅特勒公司)。

乙醇、甲醇、仲丁醇、正丙醇、异丁醇、正丁醇、异戊醇、*β*-苯乙醇等醇类,乙醛、乙缩醛、异戊醛、苯甲醛、糠醛等醛类,甲酸乙酯、乙酸乙酯、乙酸异丁酯、乙酸异戊酯、己酸乙酯、乳酸乙酯、丁二酸二乙酯等酯类以及乙酸均为色谱纯,无水氯化钠为分析纯,以上试剂购自上海安谱实验科技股份有限公司。用于实验的黄酒样品由上海石库门酿酒有限公司提供,黄酒样品类型为干型(总糖低于5 g/L),酒精度在15% vol左右。

### 1.2 实验条件

#### 1.2.1 样品前处理

将黄酒样品(1~6号)通过少量加水稀释以及添加乙醇等不同的前处理方式(见表1),改变黄酒的酒精度和挥发性组分的含量。

**表1:** 黄酒样品的前处理方式

Sample No.	Pretreatment method
1	70 mL Huangjiu+30 mL water
2	90 mL Huangjiu+10 mL water
3	100 mL Huangjiu
4	100 mL Huangjiu+2 mL ethanol
5	100 mL Huangjiu+5 mL ethanol
6	80 mL Huangjiu+20 mL 15% ethanol aqueous solution

#### 1.2.2 分析条件

酒精度和密度检测方法^[18]^:取50 mL黄酒样品,依次注入安东帕酒精分析仪和密度计的样品池,数值稳定后得到酒精度(vol)和密度值(g/cm^3^)。

挥发性风味组分的检测方法^[19]^:采用HS-GC进行检测,在20 mL顶空瓶内加入10 mL样品和3.0 g无水氯化钠,混匀后在50 ℃下平衡30 min;检测器:FID;程序升温条件:起始柱温40 ℃,保持8 min,以10 ℃/min的速率升温至220 ℃,并保持8 min;检测器温度250 ℃;载气:高纯氮,流速1.0 mL/min;采用分流进样,分流比为1∶1。

### 1.3 数据处理和分析

根据样品的稀释倍数,将每个挥发性组分的峰面积折算到稀释前的峰面积。通过1~5号不同酒精度样品的峰面积(已折算到稀释前)变化,验证乙醇基质效应对挥发性组分定量检测的影响。将对应组分的峰面积和乙醇含量作线性拟合,得到线性回归方程*y=ax+b*,以及线性回归决定系数(*R*^2^)。其中,*x*为乙醇含量,*y*为挥发性组分峰面积,*a*为斜率(即每升高或降低1% vol乙醇时,该组分峰面积的变化值)。最后,如果拟合方程呈线性相关(*R*^2^>0.8),则计算出该黄酒样品中挥发性组分峰面积在气液平衡过程中受乙醇含量影响的系数(峰面积的变化值*a*与3号黄酒原始样品中相应挥发性组分峰面积之比)。

分析过程中,每个样品重复测定3次,结果用“平均值±标准差”表示。在稀释倍数的计算过程中,需要通过密度值修正黄酒与水或乙醇混合时产生的体积变化。

## 2 结果和讨论

### 2.1 黄酒样品的稀释和检测

根据表1中的前处理方法分别加水、乙醇或乙醇水溶液对黄酒样品进行小范围稀释,使3号原始样品的酒精度和挥发性组分的稀释倍数发生变化。测量1~6号样品及15%乙醇水溶液的酒精度和密度,同时计算出1号、2号、4号、5号、6号样品的稀释倍数,结果见表2。3号原始样品经过前处理后,样品的酒精度发生变化(10.31%~18.81% vol)。同时,其他挥发性组分的含量随稀释倍数发生变化。

**表2:** 黄酒样品的理化指标及稀释倍数(*n*=3)

Sample No.	Alcohol content/ (% vol)	Density/ (g/cm^3^)	Dilution rate
1	10.31±0.02	1.0001±0.0002	1.442
2	13.22±0.03	1.0011±0.0002	1.120
3	14.71±0.01	1.0016±0.0001	1.000
4	16.43±0.02	0.9995±0.0002	1.016
5	18.81±0.02	0.9964±0.0002	1.045
6	14.76±0.03	0.9974±0.0004	0.802

Alcohol content of 15% ethanol aqueous solution is (15.11±0.02)% vol, and the density is (0.9783±0.0002) g/cm^3^.

### 2.2 乙醇含量与峰面积相关性分析

对黄酒样品进行前处理后,采用HS-GC进行黄酒挥发性组分检测。通过标准品的出峰时间确定黄酒中20种挥发性组分,结果见图1。

**图1:**
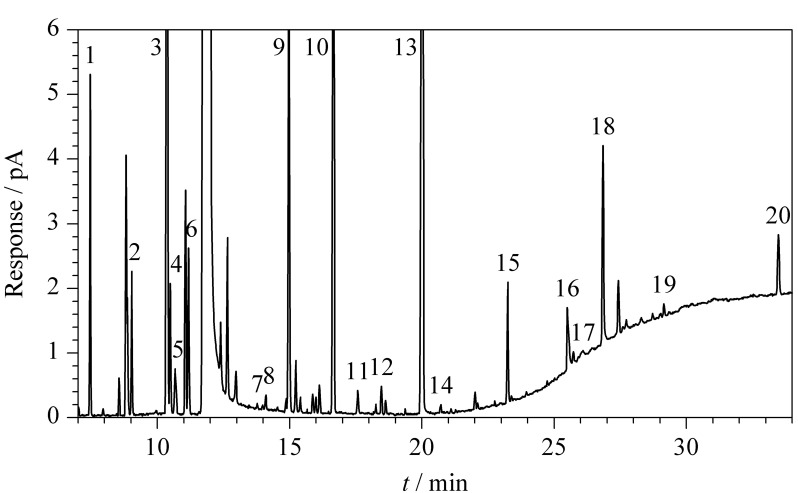
黄酒中20种挥发性组分的色谱图

以上20种挥发性组分主要分为醇、酯、醛、酸等4大类风味组分,其中正丁醇、乳酸乙酯、乙缩醛和乙酸是各类化合物中的典型风味组分。以正丁醇、乳酸乙酯、乙缩醛和乙酸为例,分析4种挥发性组分的峰面积受乙醇含量的影响及两者间的相关性(见图2)。图中以1~5号样品的酒精度为横坐标,每个样品中挥发性组分经稀释倍数折算后的峰面积为纵坐标。其中,正丁醇和乳酸乙酯的峰面积与酒精度呈负相关,*R*^2^分别为0.9349和0.8417。乙缩醛的峰面积与酒精度呈正相关(*R*^2^为0.8690),而乙酸无相关性(*R*^2^为0.0193)。醇类和酯类化合物在黄酒中的稳定性较好,在稀释、升温平衡等前处理过程中不会迅速发生化学性质的变化。

**图2:**
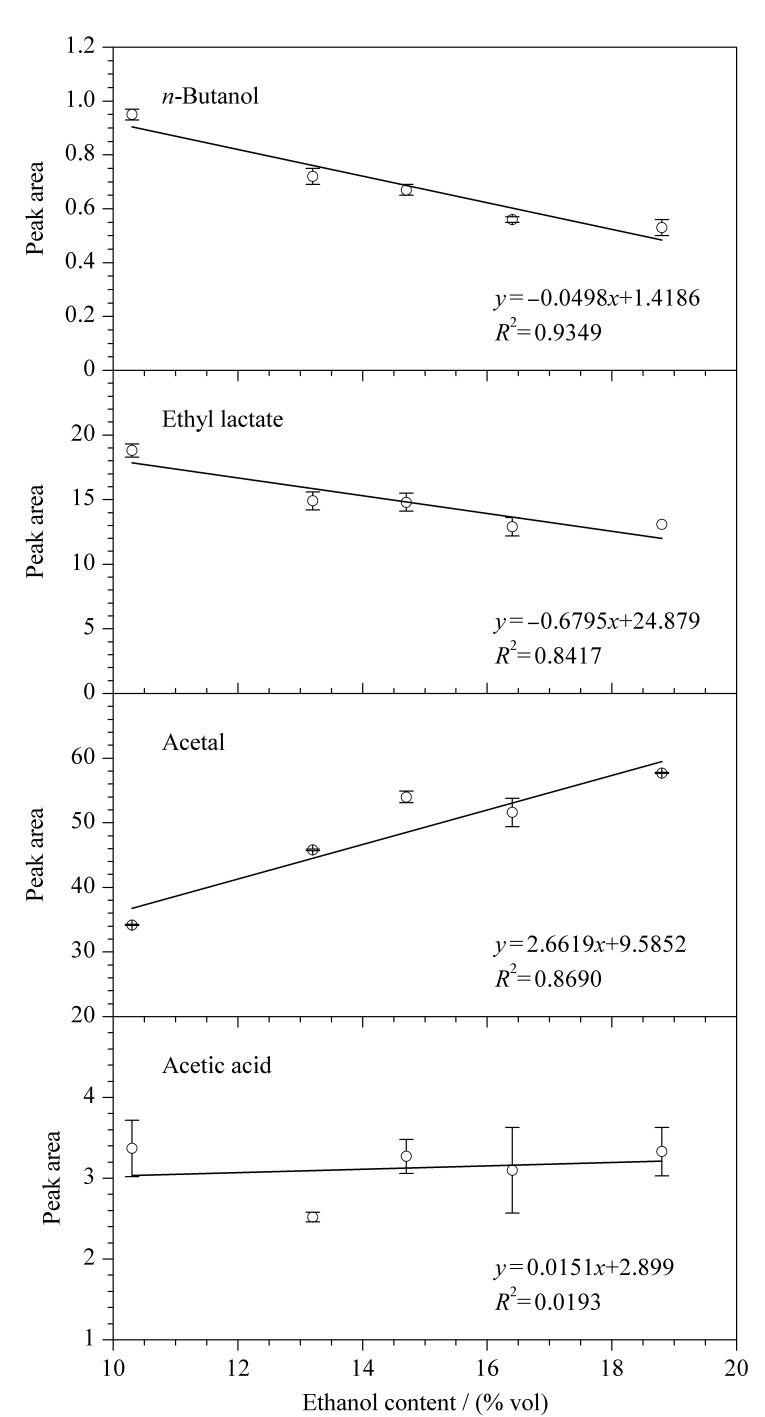
黄酒中乙醇含量与正丁醇、乳酸乙酯、乙缩醛和乙酸峰面积的相关性分析(*n*=3)

在气液两相采用顶空方法进行样品前处理时,随着黄酒样品酒精度的升高,主要溶剂乙醇的蒸汽压升高而其他溶质的蒸汽压降低,即通过顶空法检测得到的乙醇峰面积变大,但醇、酯类挥发性组分的峰面积反而减少。乙缩醛属于醛类化合物,在黄酒中的稳定性较差。当酒精度升高时,乙醇和乙醛间发生缩合反应而浓度升高,因此检测得到的峰面积也随之升高。乙酸属于弱电解质,当酒精度变化时,峰面积没有明显变化,受乙醇浓度影响较小。

### 2.3 乙醇含量对定量结果的影响

由于醇、酯、醛等挥发性组分受乙醇的影响并不相同,进一步比较黄酒中20种挥发性组分的峰面积受乙醇含量的影响程度,结果见表3。在本次乙醇含量变化范围内,共有16种化合物的峰面积与乙醇含量呈负相关,*R*^2^均大于0.8。对以上挥发性组分计算乙醇影响系数,结果为-12.4%~-2.5%,受乙醇影响程度最高的是己酸乙酯。以3号黄酒样品为例,酒精度每降低1% vol,检测得到的己酸乙酯峰面积(已折算到原浓度)反而升高12.4%,严重影响定量检测。甲醇、糠醛和乙酸与乙醇含量的相关性则较低,*R*^2^均小于0.2,峰面积没有明显变化规律。可能这3种化合物在样品前处理过程中,受乙醇基质效应、电离和化学反应等多种因素的影响。只有乙缩醛的峰面积与乙醇含量呈正相关,且*R*^2^也大于0.8,乙醇影响系数为4.9%。同时,醇、酯、醛、酸等不同挥发性组分,在气液平衡时受乙醇含量的影响程度差异较大。不同化合物的峰面积不是同比例变化,因此采用内标化合物进行校正也无法减少乙醇基质效应带来的误差。

**表3:** 20种挥发性组分受乙醇基质效应的影响程度比较(*n*=3)

No.	Compound	*R*^2^	*a*	Peak area of original sample (No. 3)	Influence coefficient/%
1	acetaldehyde	0.8652	-2.9801	117.5±1.5	-2.5
2	ethyl formate	0.9450	-0.2643	9.22±0.14	-2.9
3	ethyl acetate	0.9747	-14.872	285.0±3.7	-5.2
4	acetal	0.8690	2.6619	54.0±0.9	4.9
5	methanol	0.1133	-0.0286	3.68±0.13	/
6	isovaleraldehyde	0.9767	-0.6962	10.4±0.1	-6.7
7	isobutyl acetate	0.9613	-0.0456	0.60±0.05	-7.6
8	*sec*-butanol	0.9582	-0.1200	1.58±0.01	-7.7
9	*n*-propanol	0.8529	-1.0681	26.0±0.4	-4.1
10	isobutanol	0.9667	-13.436	182.1±2.5	-7.4
11	isoamyl acetate	0.9517	-0.1566	1.52±0.03	-10.3
12	*n*-butanol	0.9349	-0.0498	0.67±0.02	-7.5
13	isoamyl alcohol	0.9626	-33.069	315.7±4.4	-10.5
14	ethyl hexanoate	0.8672	-0.0966	0.78±0.01	-12.4
15	ethyl lactate	0.8417	-0.6795	14.8±0.7	-4.6
16	acetic acid	0.0193	0.0151	3.27±0.21	/
17	furfural	0.1447	-0.0805	1.06±0.04	/
18	benzaldehyde	0.9539	-0.1215	1.15±0.03	-10.6
19	diethyl succinate	0.9139	-0.1071	0.93±0.01	-11.5
20	*β*-phenylethanol	0.8366	-0.3499	4.62±0.31	-7.6

*R*^2^: The linear regression coefficient for ethanol content and peak area of volatile compound. *a*: The slope of the linear regression line through the given data points. /: the influence coefficient of ethanol content on the peak area of volatile components cannot be calculated because *R*^2^ is lower than 0.8 and has no correlation.

### 2.4 黄酒中乙醇基质效应的控制

通过对1~5号不同酒精度黄酒样品进行测试,发现在本次乙醇含量实验范围内(10%~19% vol),大部分化合物的蒸汽压与乙醇含量间呈负相关,乙醇的基质效应作用显著。因此,对挥发性组分采用HS-GC及气液平衡原理定量测量时,应对样品采取有效的前处理措施避免基质效应,或使不同样品受相同程度的乙醇基质效应影响。3号样品的酒精度接近15% vol,采用15% vol的乙醇水溶液进行稀释,制备得到相近乙醇含量的6号样品。经过样品前处理后,6号样品各挥发性组分含量稀释为3号样品的80.2%。通过相同条件检测6号样品,验证通过调整酒精度控制乙醇基质效应后,挥发性组分总峰面积的受影响程度,结果如图3所示。随着1~5号黄酒样品中酒精度的升高,按稀释比例折算后的挥发组分总峰面积减少。说明黄酒中酒精度升高,降低了挥发性溶质的蒸汽压,因此总色谱峰面积减少。

**图3:**
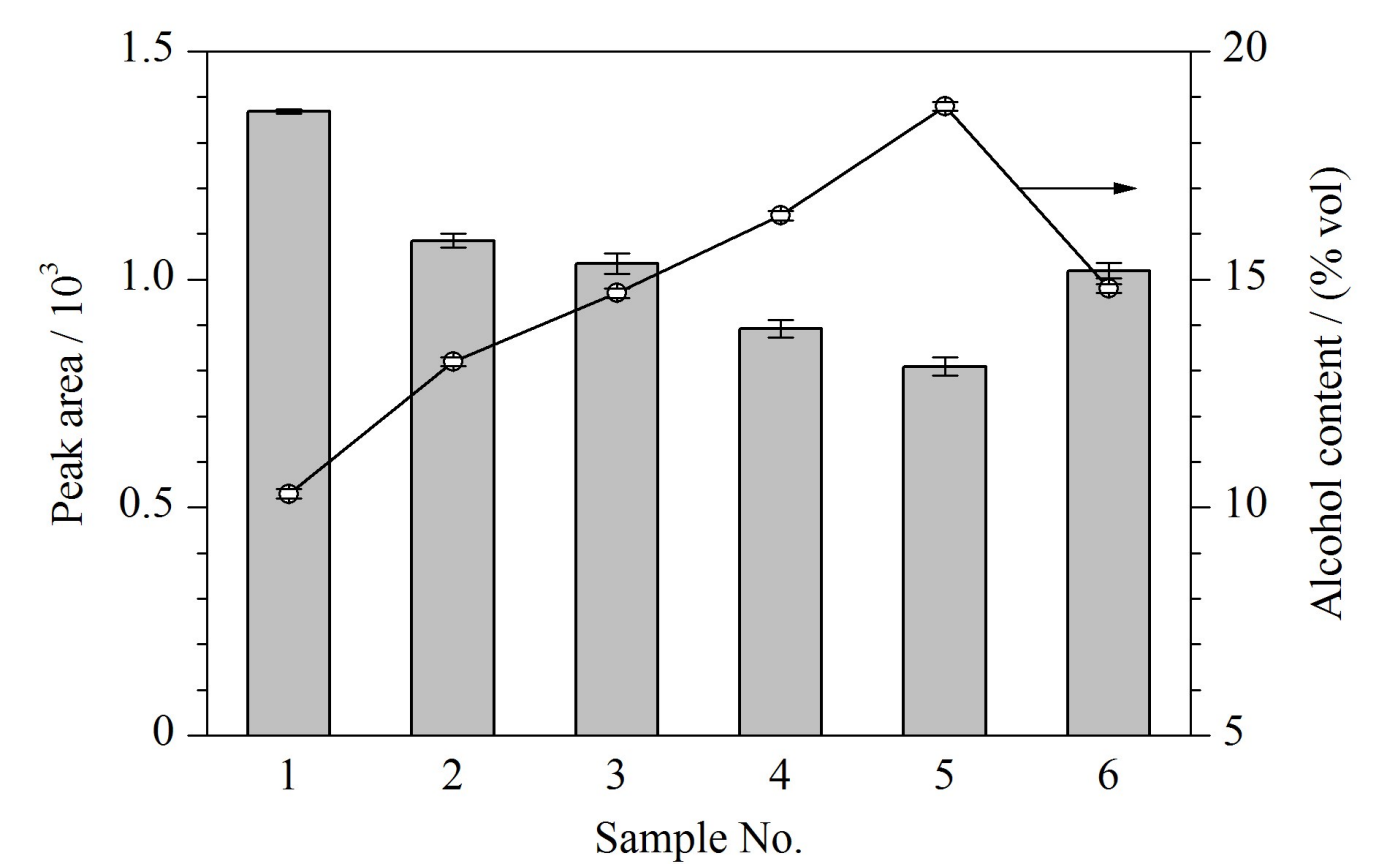
黄酒样品的酒精度和按稀释倍数折算后的总峰面积(*n*=3)

而经过稀释处理的6号样品,虽然挥发性组分浓度发生了改变,但其酒精度与3号原始黄酒样品几乎一致(分别为14.8% vol和14.7% vol),同时折算到稀释前的总峰面积也与3号原始样品接近(分别为1019和1035)。因此,将样品在较小的稀释倍数内调整至同一酒精度后,有效控制了乙醇含量差异带来的基质效应。因此不同样品中各种挥发性组分在溶液中的浓度与色谱峰面积成正比,HS-GC方法才能有效实现定量检测。

## 3 结论

黄酒的酒液作为溶剂,主成分不仅是水,还含有6%~25% vol的乙醇。而酒中溶质作为风味研究的重点,成分更为复杂。不仅有醇、酯、醛等各类微量挥发性组分,还有含量较高的糖、蛋白质和有机酸等难挥发组分。通过HS-GC开展黄酒风味组分检测,发现乙醇含量对不同挥发性组分的气液平衡存在影响,且不同组分受乙醇基质效应的影响程度存在较大差异。同时,乙缩醛等化合物在酒中的性质并不稳定,限制了大比例稀释等控制基质效应方法的使用。本研究将不同溶质浓度的样品通过较小的稀释倍数调整至同一酒精度后,不同样品中挥发性溶质在溶液中的浓度与色谱峰总面积成正比。乙醇基质效应对检测结果的干扰明显降低,定量分析的结果更加准确。

部分研究人员使用基于气液平衡原理的前处理方法对黄酒样品进行分析时,仅通过添加内标化合物进行色谱峰面积校正和定量分析。基于以上研究结论,该前处理方法不能完全消除基质效应带来的检测误差。此外,应用气体传感器或者是人工嗅闻等方法分析挥发性组分,也需要关注酒中乙醇等高浓度组分带来的基质效应。在已实施的黄酒行业检测标准中,需要对此情况进行特殊说明,避免误差的引入。此外,部分检测方法采用样品大比例稀释(稀释倍数2以上)的方法来消除基质效应,则需要关注前处理过程中组分间的物理化学反应,例如乙缩醛的缩合反应、酯类物质的水解反应等,实现不稳定挥发性组分的定量检测。
